# Polysaccharide Biosynthesis: Glycosyltransferases and Their Complexes

**DOI:** 10.3389/fpls.2021.625307

**Published:** 2021-02-19

**Authors:** Olga A. Zabotina, Ning Zhang, Richard Weerts

**Affiliations:** Roy J. Carver Department of Biochemistry, Biophysics and Molecular Biology, Iowa State University, Ames, IA, United States

**Keywords:** polysaccharide biosynthesis, glycosyltransferases, protein complexes, plant Golgi, structural organization

## Abstract

Glycosyltransferases (GTs) are enzymes that catalyze reactions attaching an activated sugar to an acceptor substrate, which may be a polysaccharide, peptide, lipid, or small molecule. In the past decade, notable progress has been made in revealing and cloning genes encoding polysaccharide-synthesizing GTs. However, the vast majority of GTs remain structurally and functionally uncharacterized. The mechanism by which they are organized in the Golgi membrane, where they synthesize complex, highly branched polysaccharide structures with high efficiency and fidelity, is also mostly unknown. This review will focus on current knowledge about plant polysaccharide-synthesizing GTs, specifically focusing on protein-protein interactions and the formation of multiprotein complexes.

## Plant Polysaccharides and Their Importance

Plant polysaccharides that constitute plant cell wall extracellular structures in the form of lignocellulosic biomass are considered to be a source of renewable energy. Polysaccharides organized in highly cross-linked networks of cell walls contribute significantly to numerous physiologically important processes such as growth (Gu and Nielsen, 2013; Xiao et al., 2016; Rui et al., 2017; Sampathkumar et al., 2019), stress protection (De Lorenzo et al., 2019; Vaahtera et al., 2019), and signal transduction (Wolf et al., 2012; Wolf, 2017; Novakovic et al., 2018).

The plant cell wall is a heterogeneous, highly diverse, and dynamic network predominantly made of different polysaccharides, a unique feature of plant cells. These polysaccharides are usually divided into three major groups: cellulose, hemicellulose, and pectin. While cellulose is a linear homopolymer composed of glucoses, hemicelluloses and pectic polysaccharides are highly branched heteropolysaccharides constructed from 15 different glycosides. The evolution of plants depended on the diversity that evolved in cell walls (Popper, 2008). Consequently, plants developed highly sophisticated and dynamic synthetic machinery to modify cell walls as needed for survival.

In the past decade, substantial advancement has been made in the understanding of cell wall polysaccharide biosynthesis, particularly, in discovering and characterizing the many genes involved in this complex process. Glycosyltransferases (GTs), the enzymes involved in the synthesis of glycans or glycosylation, are distributed among 105 gene families annotated in the CAZy database (Cantarel et al., 2009). With respect to the challenges of revealing the genes involved in the glycosylation and synthesis of complex polysaccharides, numerous studies using molecular biology, reverse genetics, and genomics have greatly advanced the understanding of the complexity of plant cell wall formation and, most notably, polysaccharide synthesis. A significant number of recent reviews and research papers describe the advances in our understanding of plant polysaccharide biosynthesis, primarily *via* genetic and reverse-genetic studies (Hao and Mohnen, 2014; Rennie and Scheller, 2014; Ebert et al., 2015; Loqué et al., 2015; Pauly and Keegstra, 2016; Amos and Mohnen, 2019; Meents et al., 2019). However, the progress in biochemical characterization of the proteins as actual players of biosynthetic processes is much slower and still lags behind more successful genetic studies. In part, the low solubility of GTs as membrane proteins and the frequent lack of suitable enzymatic assays significantly limit the progress in their functional characterization. In nature, the small stereochemical differences between sugar moieties and the diverse ways these sugars can be linked to each other create the wide diversity of oligo- and polysaccharide structures present in different types of plant cell walls. Such diversity requires a broad spectrum of suitable substrates for enzymatic assays to confirm the functionality of GTs involved in each biosynthetic process and also complicates the characterization of possible products. Despite such limitations, the structural characterization of plant GTs and their mechanisms of catalysis has become a subject of intensive research due to their essential function in plant cell wall biosynthesis and broad potential applications.

Another fundamental problem which limits our understanding of polysaccharide biosynthesis is the lack of knowledge about how GTs are organized and distributed in cell compartments. The synthesis of branched heterogeneous polysaccharides often requires the action of multiple, highly specific GTs which transfer various donor sugar substrates to oligosaccharide acceptors. It is well established that the Golgi is the organelle where most glycosylation processes take place and where the majority of GTs involved in protein and lipid post-synthetic glycosylation and complex polysaccharide synthesis are localized (Schoberer and Strasser, 2011; Sogaard et al., 2012; Ebert et al., 2015; Strasser, 2016). Another site of polysaccharide synthesis is the plasma membrane, where cellulose synthesis takes place (McFarlane et al., 2014; Kumar and Turner, 2015; Kumar et al., 2018; Turner and Kumar, 2018; Zhu et al., 2018). From the examples available to date of GTs involved in protein N-glycosylation, it has been concluded that they are organized in protein complexes and distributed across various Golgi cisternae performing specific sequential steps of glycosylation (Schoberer and Strasser, 2011; Schoberer et al., 2013; Kellokumpu et al., 2016; Strasser, 2016). Much less is known about the organization and distribution of GTs involved in polysaccharide biosynthesis.

## Glycosyltransferases, Their Mechanisms, Structural Organization, and Specificity

Solved structures for several GTs from various organisms, most of which are involved in the glycosylation of peptides, DNA, or small lipophilic molecules, have shown that most GTs have two types of catalytic domain folds, GT-A and GT-B (Lairson et al., 2008). Another type, GT-C, was also observed and proposed to be typical for GTs with multiple transmembrane helices (8–13), with specificity to lipid phosphate-activated donor sugar substrates (Lairson et al., 2008). Proteins with GT-A folding contain two tightly associated β/α/β Rossmann-like folds, forming a continuous β-sheet. Proteins with GT-B folding also have two β/α/β Rossmann-like folds. They are not tightly intertwined but instead face each other with the active site localized in between them. GT-A proteins are metal dependent, which is coordinated by the well-documented DxD motif (Wiggins and Munro, 1998), while a significant number of GT-B proteins that have so far been characterized are metal independent. In addition to these two structural folds, GTs are further divided into two distinguished groups based on whether they catalyze the formation of new covalent bonds with inverted or retained stereochemistry with respect to the donor substrate. The donor substrates are typically activated sugars such as UDP- or GDP-bound nucleotide sugars or, less frequently, phosphorylated sugars (Lairson et al., 2008). In the most common nucleotide sugars used as donor substrates, the sugar is linked to the nucleotide *via* an alpha bond. When a GT catalyzes the formation of the glycosidic linkage, attaching the sugar to the acceptor molecule *via* alpha bond, the stereochemistry is retained. When the sugar is attached *via* a beta bond, the stereochemistry is inverted. It has been demonstrated and is well accepted that the catalytic mechanism of inverting GTs is a direct displacement SN2-like reaction. In this reaction, an active site residue acts as a general base to deprotonate the acceptor, which performs a nucleophilic attack on the donor anomeric carbon (Lairson et al., 2008). On the other hand, the mechanism of retaining GTs has yet to be clarified. Currently, two preferred mechanisms have been proposed: a double displacement or a front-side single displacement mechanism (Gomez et al., 2012; Schuman et al., 2013; Albesa-Jove et al., 2015). Plant cell wall biosynthetic enzymes with both mechanisms of reaction have been found in all three structural classifications mentioned above.

**Figure 1:**
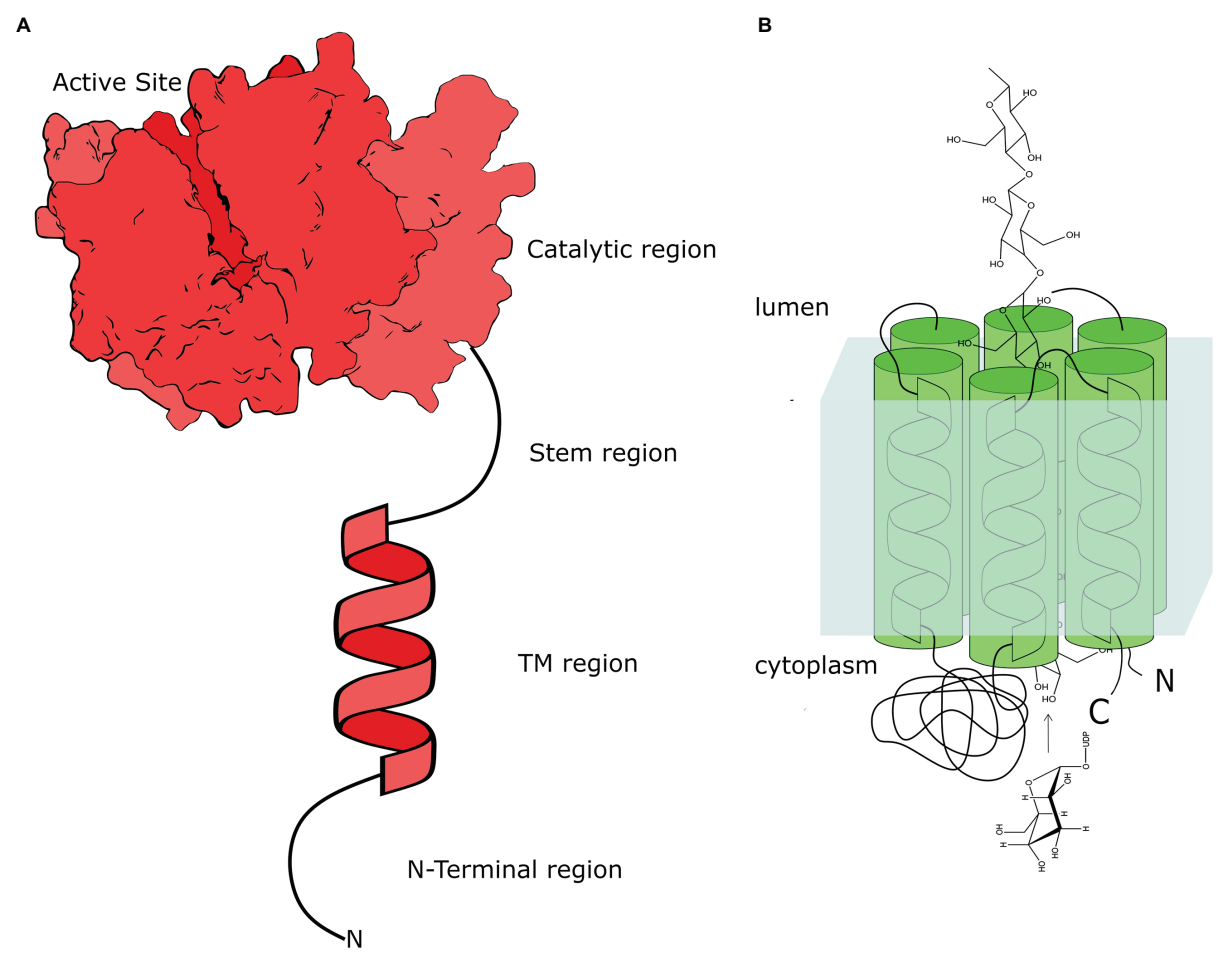
Schematic structural organization of two types of plant glycosyltransferases involved in polysaccharide biosynthesis in plant Golgi. **(A)** The structural organization of typical glycosyltransferase (GT) as a type II membrane protein with the large soluble catalytic domain attached *via* the flexible, most likely, unfolded stem region to TMD and the short N-terminus extruded out the membrane. These GTs have a single hydrophobic helix as their TMD. The actual crystal structure of XXT1 (Culbertson et al., 2018) is used here to show the catalytic domain localized in the Golgi lumen. **(B)** The structural organization of the cellulose synthase-like (CSL) proteins as the integral membrane proteins with multiple TMDs and the soluble catalytic domain that could localize either in the cytoplasm or in the Golgi lumen. Depending on the number of TMD helices spanning the membrane, the C- and N-termini of CSLs can localize on the same or opposite sides of the Golgi membrane (Davis et al., 2010).

In plants, two types of GTs are involved in polysaccharide biosynthesis (Figure 1). The first are those that have a single transmembrane domain (TMD) anchoring the catalytic domain of GTs to the membrane. The other type of GT has multiple TMDs. Usually, six to eight helices span the membrane multiple times, forming an open channel across the membrane. Cellulose synthase (Ces) and Cellulose synthase-like (CSL) are integral membrane proteins and belong to CAZy family GT2 (Cantarel et al., 2009). They have multiple TMDs spanning the Golgi membrane (or plasma membrane, in the case of Ces) multiple times and a large soluble catalytic domain localized outside the membrane. It has been demonstrated that CSL proteins are involved in synthesizing linear polysaccharides, such as the glucan backbone in xyloglucan (Cocuron et al., 2007), mixed glucans in monocots (Doblin et al., 2009), and glucomannans and mannans (Liepman et al., 2007). These proteins likely act *via* a mechanism similar to that of cellulose synthases CesA (Morgan et al., 2013; Slabaugh et al., 2014; McNamara et al., 2015; Purushotham et al., 2020), where the substrates are taken from the cytosol and the synthesized polysaccharide are translocated through the membrane into the Golgi lumen. However, mannan synthase CSLA9 was reported to have its active site on the luminal side of the Golgi membrane (Davis et al., 2010), suggesting the existence of different modes of action among these proteins.

However, two groups recently discovered that some members of the CSLG superfamily catalyze the transfer of a glucuronic moiety from UDP-GlcA onto triterpenoid aglycones, glycosylating saponins during their biosynthetic pathway (Chung et al., 2020; Jozwiak et al., 2020). It is interesting that spinach CSLG transiently expressed in *Nicotiana benthamiana* leaves was localized in the ER, not in the Golgi. The template-based modeling showed the presence of six TMDs and predicted three channels that could form the path for triterpenoid acceptors into the active site of the enzyme, with an additional channel for the product exit (Jozwiak et al., 2020). However, it is unclear whether CSLG translocates the product from one side of the ER membrane to the other, as was shown for the CesA enzymes. These unexpected findings demonstrate the ability of the CSL proteins to evolve new substrate specificities with a limited number of amino acid substitutions in the active site and open the door to similar discoveries.

Other GTs involved in cell wall polysaccharide biosynthesis are type II membrane proteins and reside in the Golgi (Driouich et al., 2012; Sogaard et al., 2012; Parsons et al., 2019). These GTs possess a short N-terminus extruding to the cytosolic side of the Golgi membrane, a single helical TMD, a flexible unfolded stem region, and a soluble catalytic domain localized in the Golgi lumen (Oikawa et al., 2013), though the presence of GTs without the predicted TMD has also been reported (Kong et al., 2011). To date, only two plant GTs involved in cell wall biosynthesis have been structurally characterized (Rocha et al., 2016; Urbanowicz et al., 2017; Culbertson et al., 2018). The lack of structural data for most plant GTs is a significant gap in our current knowledge about polysaccharide biosynthesis. In addition, the lack of knowledge about protein-protein interactions and the involvement of TMDs in these interactions impact our understanding of the mechanisms of polysaccharide biosynthesis and impair advances in fully understanding plant cell wall formation and its regulation.

## Cellulose Synthases Form Complexes Localized in the Plasma Membrane

Cellulose biosynthesis in the plant plasma membrane is the most well-understood process of plant polysaccharide biosynthesis. Using electron microscopy, structures responsible for cellulose synthesis have been identified in many organisms (Brown, 1996; Kimura et al., 1999). Hexagonal structures with sixfold symmetry, called rosettes, were detected in many lower and higher plants (Brown, 1996; Delmer et al., 2001). It was also revealed that rosettes are first assembled in the Golgi and then transported to the plasma membrane (Haigler and Brown, 1986). Later, numerous genetic and biochemical studies demonstrated that the subfamily of CesA genes is responsible for cellulose biosynthesis, and there are two distinguished groups of CesA involved in the synthesis of primary and secondary cell wall cellulose (Doblin et al., 2002; Persson et al., 2007; Kumar and Turner, 2015; Kumar et al., 2018; Turner and Kumar, 2018). CesA synthesizes a single glucan chain and translocates it through a channel formed by its TMD (Bi et al., 2015; Purushotham et al., 2020). Most plants express several CesA isoforms required for the synthesis of cellulose during primary and secondary cell wall formation (McFarlane et al., 2014). Although *in vitro* studies demonstrated that the singular CesA8 from poplar (Purushotham et al., 2016) was able to synthesize cellulose fibers which resembled plant cellulose microfibrils (CMF), the formation of cellulose synthase complexes (CSC) is likely required to synthesize crystalline CMF *in planta* (Purushotham et al., 2020). Plant CesAs have three specific domains that mediate their oligomerization: (1) the extended cytosolic N-termini with a RING-like domain close to the N-terminus, (2) the plant conserved region (PCR) localized in catalytic CesA domain close to the membrane side, and (3) the class-specific region which follows an evolutionarily conserved substrate-binding motif near the active site (Vergara and Carpita, 2001; Somerville, 2006; Slabaugh et al., 2014).

The understanding of the compositional and stoichiometric organization of CSC evolved through vast detailed analyses of these rosettes in multiple plant species using diverse approaches (Delmer et al., 2001; Doblin et al., 2002; Persson et al., 2007; Li et al., 2013; Sampathkumar et al., 2013; McFarlane et al., 2014; Watanabe et al., 2015). The understanding is that, in the plasma membrane, CesAs are assembled in sixfold symmetric CSCs which can consist of hexamers of either trimers, tetramers, or hexamers, thus composing rosettes with 18, 24, or 36 CesAs (Jarvis, 2013; Nixon et al., 2016). However, recent studies do not support the notion of 36 CesA present in a single complex or the formation of microfibrils composed of 36 cellulose chains. Thus, recent structural analysis of CesA1 catalytic domains has suggested that CesA can form trimeric structures, (Vandavasi et al., 2016) not homodimers as was previously believed (Olek et al., 2014). More solid evidence of such active homotrimeric CesA complexes in plants was produced recently by Zimmer’s group using recombinant full poplar CesA8 isoforms and cryo-electron microscopy (Purushotham et al., 2020). This groundbreaking work revealed that the CesA8 homotrimeric complex is stabilized by cytosolic PCRs and a helical exchange within the transmembrane-localized segments. The complex forms three channels occupied by nascent cellulose polymers, where a secretion mechanism steers the glucan chains toward a common exit point, likely facilitating the formation of a protofibril. The protein N-terminal domains are assembled into a cytosolic stalk that, in turn, interacts with a microtubule-tethering protein cellulose synthase interactive (Zhu et al., 2018), and this interaction most likely controls CesA localization (Purushotham et al., 2020). These data corroborate the earlier proposed CSC rosette organization (Nixon et al., 2016; Vandavasi et al., 2016) and the notion that each CSC produces CMFs composed of 18 chains, which was also suggested by modeling studies (Nixon et al., 2016; Kubicki et al., 2018).

## Golgi Localized Glycosyltransferases are Organized in Multiprotein Complexes

Although plant Golgi stacks are unique in their ability to synthesize and secrete massive amounts of complex polysaccharides to the cell wall, there nonetheless appears to be a broad conservation of mechanisms for protein transport and Golgi organization across various species. Thus, knowledge available regarding protein glycosylation in eukaryotic cells and, specifically, about the steady-state distribution of GTs involved in this process provides insight into the fundamental nature of the glycosylation process in the plant Golgi apparatus (de Graffenried and Bertozzi, 2004; Kellokumpu et al., 2016), and the polysaccharide-synthesizing GTs’ distribution within the Golgi may potentially follow suit. All of the plant Golgi-localized glycan-processing GTs, including GTs involved in polysaccharide synthesis, are type II membrane proteins, with a short cytosolic N-terminus and a TMD linked to their catalytic domain *via* a stem region. These features together are referred to as the CTS region, which seems to contain all information required for protein targeting to the Golgi and its sub-compartments (Saint-Jore-Dupas et al., 2004, 2006; Schoberer et al., 2009, 2013).

It has been confirmed that N-glycosylation of proteins in the Golgi is performed by multiprotein complexes (Hassinen et al., 2010; Kellokumpu et al., 2016; Khoder-Agha et al., 2019). In mammalian and plant cells, several types of Golgi-localized GTs and glycosidases, which are organized in heterooligomers, function sequentially in different Golgi sub-compartments. Such sequential distribution of GTs, where the enzymes functioning at the early steps of N-glycan processing localize in the cis- and medial-Golgi cisternae and the enzymes involved in the later steps localize in the trans-Golgi and the trans-Golgi network, likely promotes the reproducibility of the branched glycan structures synthesized within the Golgi (Hassinen et al., 2010; Schoberer and Strasser, 2011; Kellokumpu et al., 2016; Strasser, 2016). In *Arabidopsis*, several N-glycan-processing enzymes also form homodimers and heterodimers (Schoberer et al., 2013). Thus, the processing enzymes Golgi α-mannosidase I (MNS1), N-acetylglucosaminyltransferase I (GnTI), Golgi α-mannosidase II (GMII), and xylosyltransferase (XylT), which are involved in early N-glycan processing steps, form homodimers and heterodimers in cis- and medial-Golgi cisternae. In the trans-Golgi, the enzymes GALT1 and FUT13 form heterodimers with GMII during the later steps of glycan processing. These results suggest that the modification of N-glycans may depend on the distinct spatial localization of multiprotein enzyme complexes involved in this process.

The similarity between polysaccharide synthesis and N-glycosylation also extends to the existence of homodimers and heterocomplexes of biosynthetic enzymes (Atmodjo et al., 2011; Chou et al., 2012, 2015; Oikawa et al., 2013; Lund et al., 2015; Jiang et al., 2016) that appear to be differentially distributed throughout the Golgi cisternae (Chevalier et al., 2010; Parsons et al., 2019). Distinctions between protein N-glycosylation and polysaccharide synthesis, however, indicate even more complex biosynthetic machinery for cell wall components than for N-glycosylation. First, these polysaccharides are long polymers with backbones made up of regular sequences of one or, at most, two different monosaccharides, which are then decorated with diverse side chains, whereas the glycans on glycoproteins have branched structures without a regularly patterned backbone. This requires that, in addition to the GTs that form branched structures, the plant Golgi must house additional GTs that carry out backbone synthesis. Specific CSLs have been shown to be involved in the synthesis of linear glycans, such as the glucan backbone in xyloglucan, mixed linkage glucans, and glucomannans (Liepman et al., 2007; Doblin et al., 2009; Davis et al., 2010). A second distinction of cell wall polysaccharide synthesis from protein N-glycosylation is the mechanism of substrate attachment. Proteins undergoing glycosylation are frequently anchored to the Golgi membrane and so might be localized nearby and be readily available for action by GTs, which are also integrated into the membrane. By contrast, membrane anchors have not yet been identified within polysaccharides, so hypothetically it is possible that polysaccharides undergoing elongation and modification are attached to the binding side of the enzymes involved in backbone synthesis for most of the process. A third unique feature of cell wall polysaccharide biosynthesis is that long linear polysaccharides typically are not soluble (Scheller and Ulvskov, 2010; Amos and Mohnen, 2019), so side chains attached to the backbone are necessary to prevent linear glycans from aggregating *via* hydrogen bonds and precipitating inside the Golgi lumen. Therefore, the GTs involved in adding side chains have to be proximate to the CSL enzymes that synthesize the polysaccharide backbone in order to ensure the solubility of the elongated polysaccharide. This requirement can be met if biosynthetic enzymes synthesizing the backbone and the modifying GTs are all organized together in space, potentially within multienzyme complexes.

Despite the importance of plant cell wall polysaccharides in various processes critical to plant development and survival, the functional organization of polysaccharide-synthesizing GTs in the Golgi remains largely unknown. It has been proposed earlier that GTs form multienzyme complexes, which seems critical to synthesize highly complex, frequently branched polysaccharide structures (Keegstra, 2010). The most recent studies of protein-protein interactions among various polysaccharide-synthesizing GTs confirmed the formation of homodimers and heterodimers localized in the Golgi membrane (Zeng et al., 2010; Atmodjo et al., 2011; Oikawa et al., 2013; Lao et al., 2014). These new findings suggest that protein-protein interactions could be a general principle of organization of GTs involved in polysaccharide synthesis in the Golgi (Oikawa et al., 2013). For example, GAUT1, a homogalacturonan synthesizing α-1,4-galacturonosyltransferase, has been reported to interact with GAUT7, although the enzymatic activity of the latter has not been demonstrated (Sterling et al., 2006; Atmodjo et al., 2011). The study of Atmodjo et al. (2011) demonstrated that matured GAUT1 lacks a TMD due to proteolytic processing and is anchored to the Golgi membrane through covalent bonding with GAUT7. Another most recent study of the GAUT proteins expressed in *Arabidopsis* pollen revealed that the GAUT5 and GAUT6 proteins also anchor GAUT1 to the Golgi apparatus, thus increasing the number of possible GAUT heterodimers involved in the synthesis of homogalacturonan (Lund et al., 2020). This latter study demonstrated that the lack of all three anchor proteins, GAUT5, GAUT6, and GAUT7, severely impacted pollen growth, most likely due to the failed delivery of GAUT1 to the Golgi to support homogalacturonan synthesis. Another example is the interaction of two arabinosyltransferases, ARAD1 (ARABINAN DEFICIENT 1) and ARAD2 (Harholt et al., 2006, 2012), involved in synthesis of the arabinan side chains in rhamnogalacturonan I, the pectin polysaccharides in *Arabidopsis* cell walls. The ARAD1 protein was shown to form homodimers and heterodimers with its homologous protein ARAD2 when both are heterologously co-expressed in *N. benthamiana*. The authors used bimolecular fluorescence complementation (BiFC) and Förster resonance energy transfer to confirm protein-protein interactions between these proteins (Harholt et al., 2012). Current research in wheat cell wall biosynthesis suggests that another multiprotein complex functions in the biosynthesis of glucuronoarbinoxylan (GAX; Zeng et al., 2008, 2010). Three proteins, TaGT43-4, TaGT47-13, and TaGT75-4, were demonstrated to be involved in GAX biosynthesis. Results from a co-immunoprecipitation assay performed using these proteins and antibodies against TaGT43-4 demonstrated that TaGT43-4 interacts with TaGT47-13 and TaGT75-4. The pulled-down protein complex was confirmed to be enzymatically active, synthesizing arabinosylated and glucuronidated xylan oligosaccharides with a regular pattern. A study by Dilokpimol et al. (2014) revealed that two *Arabidopsis* galactosyltransferases, AtGALT29A and AtGALT31A, involved in the synthesis of arabinogalactan in arabinogalactan proteins, form heterocomplexes localized in the Golgi. In *in vitro* assays, the activity, in regards to galactose incorporation into polysaccharide chains, was higher in the case of heterocomplexes than with individual enzymes. Another recent study demonstrated that the *Asparagus* Irregular Xylem (IRX) 9, IRX10, and IRX14A proteins form multiprotein complexes responsible for xylan backbone synthesis in the Golgi (Zeng et al., 2016). The authors proposed that these proteins are organized in a large single complex where catalytically active IRX10 is indirectly anchored to the Golgi membrane *via* a heterotrimeric complex formed of IRX9 and IRX14A. Using BiFC assays, the authors observed the formation of homodimers and heterodimers among these proteins and concluded that the IRX complex could potentially include three homodimers (Zeng et al., 2016).

**Figure 2:**
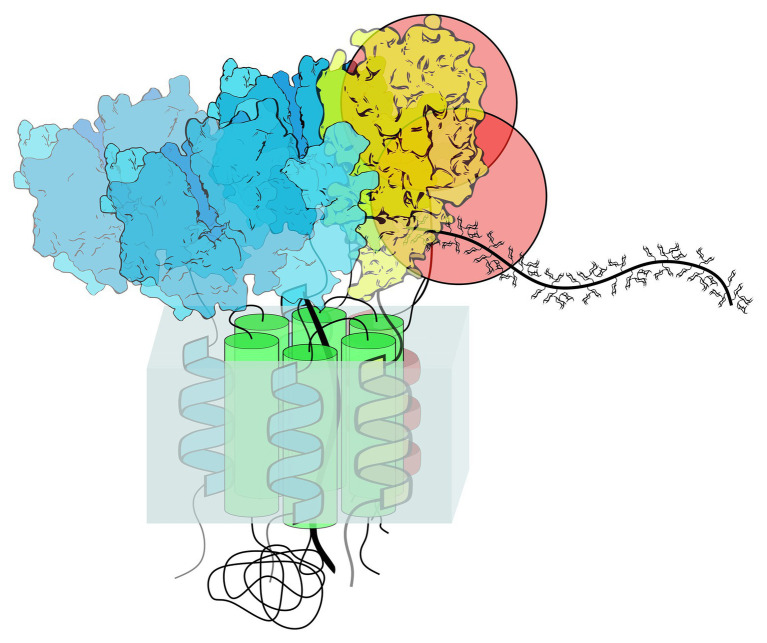
Hypothetical organization of the xyloglucan synthesizing complex. Seven glycosyltransferases (GTs) have been shown to interact in the *Arabidopsis* Golgi, forming a multiprotein complex. It is hypothesized that GTs involved in the synthesis of xyloglucan side chains are organized around the CSLC protein that synthesizes the glucan backbone. The CSLC protein has a catalytic domain localized in the cytoplasm, where it adds glucose to the elongating glucan chain, moving the chain into the Golgi lumen through the pore formed by the six TMD helices (green). The other six GTs [three blue xylosyltransferases (XXT1, XXT2, and XXT5), two red galactosyltransferases (MUR3, XLT2), and yellow fucosyltransferase (FUT1)] interact with the TMD of the CSLC protein *via* their TMDs; in addition, the GTs interact with each other *via* catalytic domains. The actual crystal structures of XXT1, XXT2, XXT5, and FUT1 (Urbanowicz et al., 2017; Culbertson et al., 2018) are depicted here. Since the structures for MUR3 and XLT2 are not solved, these GTs are depicted as red spheres.

**Figure 3:**
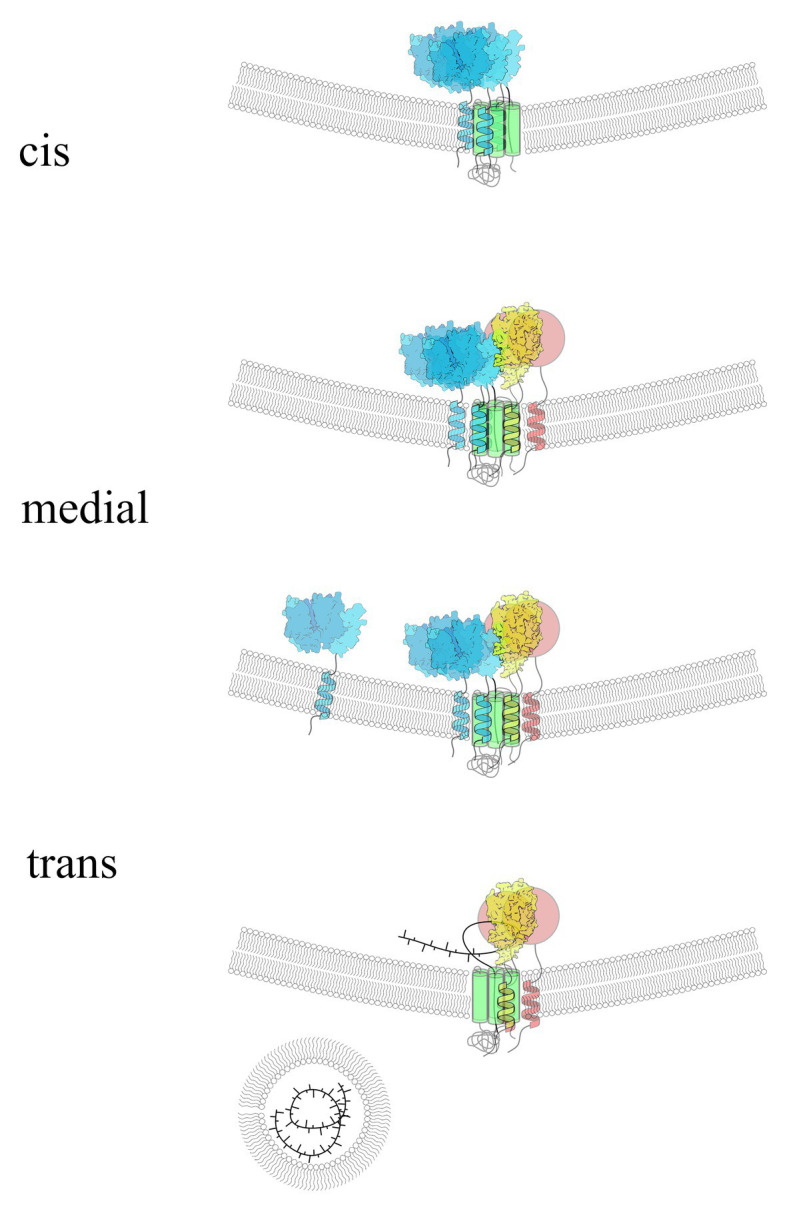
Hypothetical presentation of xyloglucan synthesizing complex organization and its compositional dynamics in different Golgi cisternae. The colors and structural organizations are identical to those shown in Figure 2. CSL (green) and three XXTs (blue) form the first stage of the complex to synthesize the glucan backbone and xylosylate it as the first step in xyloglucan biosynthesis. Later in Golgi maturation, the other three glycosyltransferases (GTs) are added to the complex to finish the side chain synthesis, while CSL and XXTs continue to elongate and xylosylate the backbone. Perhaps XXTs can leave the complex at the later stages of synthesis; however, the backbone continues to be attached to CSL, assisting MUR3, XLT2, and FUT1 to finalize the synthesis of complete polysaccharide structure. When the complex reaches the trans-Golgi, the polysaccharide is released *via* currently unknown mechanism and gets packed into transport vesicles to be delivered to the cell surface. The hypothetical distribution of xyloglucan-synthesizing GTs depicted here is based on multiple studies of protein-protein interactions among these proteins and on data from electron microscopy studies of GT localization within different Golgi cisternae.

It was proposed that Golgi-localized GTs involved in polysaccharide biosynthesis could also be distributed along different Golgi cisternae according to particular steps in the branching of polysaccharides (Zhang and Staehelin, 1992; Chevalier et al., 2010; Parsons et al., 2019). This was based on detected localizations of some branching enzymes involved in xyloglucan biosynthesis that are co-localized in medial- and trans-Golgi cisternae along with the products of their biosynthetic action (Parsons et al., 2019). Studies from our group using BiFC assays combined with flow cytometry, immunoprecipitation, and *in vitro* pull-down assays showed that the protein Cellulose Synthase Like C4 (CSLC4), which synthesizes the glucan backbone in xyloglucan, and six other proteins [three xyloglucan xylosyltransferases (XXT1, XXT2, and XXT5), two galactosyltransferases (MUR3 and XLT2), and fucosyltransferase (FUT1)] involved in the synthesis of side chains in xyloglucan form homodimers and heterocomplexes (Chou et al., 2012, 2015; Lund et al., 2015). It can be argued that the attachment of the first monosaccharides in polysaccharide side chains would be enough to produce long polymers that can stay soluble and be further branched later in their movement through Golgi stacks. This is possible in the case of some polysaccharides that do not have long side chains, like galactomannans, or in the case of non-branched mixed glucans, glucomannans, and mannans. However, the elongation of linear backbones without simultaneous branching would most likely quickly terminate synthesis due to a drop in their solubility. Thus, in the case of xyloglucan, this sequential attachment of monosaccharides in its side chains most likely occurs within the same multiprotein complex and not by the action of distinct heterodimers or small heterocomplexes. First, it was shown that xyloglucan molecules that lack Gal-Fuc saccharides attached to xylose decreases growth in *Arabidopsis* knock-out mutants. It was proposed that the reason for growth inhibition was the lower solubility of such partially branched xyloglucan in comparison with the fully synthesized polysaccharide (Kong et al., 2015). The authors proposed a model that invokes a dysfunctional xyloglucan structure as the most direct cause of growth defects of mutant plants carrying loss-of-function mutations in galactosyltransferase MUR3. Second, studies of protein-protein interactions among xyloglucan-synthesizing GTs demonstrated strong interactions between glucan synthase CSL, the enzymes XXT1, XXT2, and XXT5 involved in the first step of glucan backbone branching, and the GTs involved in the next steps of side chain synthesis, MUR3 and FUT1 (Chou et al., 2012, 2015; Lund et al., 2015). Currently, we do not know enough to rule out any possible scenarios in GT distribution along Golgi stacks and their order of action in synthesizing complete structures of the branched polysaccharide which is delivered to the cell surface. It is likely that different modes exist for different polysaccharides, depending on their branching complexity, the number and types of GTs involved, and the solubility of partially synthesized molecules. Thus, in the case of xyloglucan in *Arabidopsis*, seven different GTs are required to accomplish the synthesis of a completely branched polysaccharide (Figure 2). The number and the type of GTs can be slightly different in different plant species that have xyloglucans with variations in their side chain monosaccharide composition. We can hypothesize that glucan synthase continues an elongated glucan backbone throughout the whole time period of Golgi maturation. Xylosyltransferases form initial complexes with glucan synthase at the very beginning of synthesis initiation and continue to add xyloses to the newly synthesized glucan. Two galactosyltransferases known to be specific to the position of xyloses on the backbone likely get added to the complex in later Golgi cisternae, likely together with fucosyltransferase to complete the side chains of nascent xyloglucan, which can still be elongated by glucan synthase and xylosyltransferases (Figure 3). How this process terminates is an open question. It is possible that the complex disassembles, releasing completely synthesized branched xyloglucan, or a glucan-specific hydrolase added in the trans-Golgi cleaves the glucan backbone, releasing the branched part of the molecule.

It has been shown that some GTs function as homodimers or heterodimers while being less active in monomeric form (Hassinen et al., 2010; Kellokumpu et al., 2016). Judging from the few examples reported to date, the actual enzymatic activity of most plant polysaccharide-synthesizing GTs does not depend on being in heterocomplexes. Most polysaccharide-synthesizing GTs characterized so far can act individually in solution, catalyzing sugar transfer onto oligosaccharides if the correct acceptor is provided. So far, there are few examples of such obligatory oligomerization. One example is the plant GAUT1–GAUT7 heterodimer (Atmodjo et al., 2011), in which GAUT1 associates with GAUT7 after proteolytic cleavage of its N-terminal TMD in order to be anchored in the Golgi. The study of the GAUT1–GAUT7 complex involved in the synthesis of homogalacturonan demonstrated that this heterodimer, where GAUT1 carries the enzymatic activity and GAUT7 serves as an anchor for GAUT1, has two distinct phases, a slow phase of homogalacturonan initiation *de novo* followed by fast polysaccharide chain elongation (Amos and Mohnen, 2019). The authors proposed the two-phase distributive elongation model as an alternative to the processive GT mode of action. They suggested that, in addition to anchoring GAUT1 to the Golgi membrane, the GAUT7 protein functions as the binding site for the elongating homogalacturonan chain and directs it to the GAUT1 active site by forming the grove between the two proteins. Currently, it is unknown whether the other two anchor proteins, GAUT5 and GAUT6, also act in a similar manner, assisting the GAUT1 enzyme. Studies of *Asparagus* IRX proteins involved in xylan synthesis also suggested that IRX10 possibly requires interaction with IRX9 and IRX14A to be anchored to the Golgi, although it was not demonstrated whether the activity of these proteins depends on the presence of others (Zeng et al., 2016). When the CSLC4 protein (Cocuron et al., 2007) was co-expressed with XXT1 in *Pichia* cells, it was able to synthesize longer glucan chains in comparison to the expression of CSLC4 alone. Although the glucan chain was not xylosylated due to the absence of UDP-Xyl in *Pichia*, these results suggested that XXT1 may assist CSLC4 function through direct or indirect interactions.

Protein in multiprotein complexes can be held together *via* various interacting surfaces. There are examples of such interactions between different GT domains, such as hydrophobic TMDs, soluble catalytic domains, stem regions, or short cytosolic N-termini (Atmodjo et al., 2011; Harrus et al., 2018; Khoder-Agha et al., 2019; Purushotham et al., 2020). Less information about protein domains involved in protein-protein interactions is available for polysaccharide-synthesizing GT complexes. For example, in the case of GTs involved in the synthesis of side chains in xyloglucan, all six enzymes were shown to interact when expressed and purified without their predicted TMDs using pull-down assays (Chou et al., 2012, 2015). This suggests that they can interact *via* catalytic domains localized in the Golgi lumen. It is plausible that the xyloglucan-synthesizing complex is held together *via* hydrophobic interactions between TMD domains of glucan synthase and the branching GT within the Golgi membrane; in addition, the soluble catalytic domains of branching GTs interact with each other in the Golgi lumen. Bringing together the catalytic domains of GTs in the Golgi lumen *via* interaction might assist in moving the elongating glucan backbone along their active sites. A study of protein-protein interactions between GAUT1 and GAUT7 showed that they interact through their catalytic domains due to lack of a TMD in mature GAUT1, and this heterocomplex is held together *via* covalent and non-covalent interactions (Atmodjo et al., 2011).

## Structural Studies Demonstrate Oligomerization of Plant GTs

Only two crystal structures are currently available for plant GTs involved in cell wall non-cellulosic polysaccharide biosynthesis. Both structurally characterized GTs are *Arabidopsis* proteins involved in xyloglucan side chain synthesis. The first is XXT1, which initiates side chains on the non-branched glucan backbone during xyloglucan biosynthesis (Cavalier and Keegstra, 2006; Cavalier et al., 2008). The other is FUT1, which attaches the terminal fucose to finish the trisaccharide attached to the glucan backbone in xyloglucan (Perrin et al., 1999; Faik et al., 2000).

*Arabidopsis* FUT1 was the first plant polysaccharide-synthesizing GT structurally characterized using X-ray crystallography. Two groups have independently solved the structure of FUT1 (Rocha et al., 2016; Urbanowicz et al., 2017). Both groups reported the oligomerization of the recombinant FUT1 without a TMD in solution observed during purification and in the final crystals. Rocha et al. (2016) obtained a crystal with four molecules of FUT1 in an asymmetric unit, which corroborated their earlier findings that FUT1 behaves as a noncovalent homodimer in solution (Ciceron et al., 2016). Urbanowicz et al. (2017) reported two structures, where the two molecules of FUT1–GDP complex were present in the asymmetric unit, whereas another crystal contained four molecules of FUT1–XXLG complex (Urbanowicz et al., 2017).

Later, the crystal structure of XXT1 was solved by our group (Culbertson et al., 2018). The XXT1 protein, lacking a TMD, also behaves as a noncovalent homodimer in solution and presented as a symmetric homodimer in the crystals obtained. Considering that GTs frequently tend to oligomerize in solution, one can argue that the homodimerization of FUT1 and XXT1 observed in structural studies is an *in vitro* artifact. However, analyses of available structural data for numerous GTs involved in various glycosylation processes and present in oligomeric form in their crystals show that many GTs actually form biologically relevant dimers (Harrus et al., 2018). Studies of protein-protein interactions in living plant cells corroborated the reported behavior of the XXT1 and FUT1 proteins observed in solution. Homodimers for two XXTs, FUT1, and CSLC4 were detected using BiFC assay in *Arabidopsis* (Chou et al., 2015) and a reversible Renilla luciferase protein complementation assay in *N. benthamiana* (Lund et al., 2015). From the latter’s cell biology studies, it was concluded that GTs involved in xyloglucan biosynthesis can form homodimers and heterodimers *in vivo*, though competition assays reveal that some proteins form stronger heterodimers than homodimers (Chou et al., 2012, 2015). Although an XXT1 homodimer was not detected in the BiFC study, it is possible that it can still be formed transiently before XXT1 gets to interact with XXT2 to form a more stable complex. These results support earlier conclusions made from reverse-genetic study of *Arabidopsis* XXTs (Zabotina et al., 2012). There it was proposed that the heterodimers XXT2–XXT5 and XXT1–XXT2 are key players during xyloglucan biosynthesis in *Arabidopsis* plants. Indeed when we used the structure of the XXT1 homodimer to generate homology models of XXT2 and XXT5, we found that a large number of identical residues in all three proteins are involved in protein-protein interactions holding their homodimers (Zabotina lab, not published). Furthermore, for XXT1–XXT1 and XXT2–XXT2 homodimers, all residues involved in ionic or hydrogen bonding between the two proteins were identical, and in XXT5–XXT5 only one pair of amino acids was different from the pair at the same position in the homodimers of XXT1 and XXT2. These observations suggest that the XXT proteins are interchangeable and can readily form homodimers or heterodimers *via* the same interaction surfaces. At this point, it is difficult to propose what implications such high fidelity of interacting residues in XXT proteins has on the stoichiometry of the whole xyloglucan-synthesizing complex in the Golgi and the biological relevance of the XXT1 homodimer, but there is likely certain flexibility in their compositional dynamics.

## Models for Distinct Sub-Golgi Localization of Glycosyltransferases

GT sorting, concentration, and assembly into complexes in specific spatial locations necessarily depend on the nature of protein transport through the Golgi (Munro, 1998; Pelham and Rothman, 2000; Tu and Banfield, 2010). Two main models of protein trafficking are currently intensely discussed: “anterograde vesicular transport” and “cisternal maturation” (Opat et al., 2001; Staehelin and Kang, 2008; Donohoe et al., 2013). The cisternal maturation model depends on the efficient retrograde transport of cis- and medial-Golgi proteins from the late Golgi cisternae back to the earlier cisternae to maintain the steady-state spatial distribution of GTs across the Golgi stack. Selective enrichment of late-Golgi-resident proteins could also be achieved by sustained and very efficient recycling of these proteins from post-Golgi compartments, like the TGN, back to trans-Golgi cisternae. On another hand, the vesicular transport model requires continuous trafficking of GTs to specific Golgi cisternae in combination with a mechanism to retain them by preventing their anterograde transport. Data from plant cells appear to be most consistent with the cisternal maturation model (Hawes et al., 2010; Donohoe et al., 2013).

The mechanisms that account for the specific spatial distributions of polysaccharide biosynthetic enzymes throughout the Golgi cisternae are unknown despite the fact that, as noted, this feature of the biosynthetic system is integral to determining the structure of mature cell wall components. Current knowledge primarily derives from studies of protein glycosylation. Four different models have been proposed for the sub-Golgi distribution of GTs involved in glycoprotein modification (Welch and Munro, 2019): (1) oligomerization or “kin recognition” (Opat et al., 2000; Andersson-Gunneras et al., 2006; Hassinen et al., 2010), (2) the “bilayer thickness” model (Saint-Jore-Dupas et al., 2006), (3) the rapid partitioning model (Patterson et al., 2008), and (4) sub-targeting *via* information encoded in the cytoplasmic tails of GTs (Uliana et al., 2006). On the other hand, despite intensive studies of N-glycosylation in plants over the past decades, the underlying mechanisms of spatial localization are still widely unsolved. The data that are available emphasize that sorting/partitioning of proteins and lipids into different Golgi cisternae is a highly interdependent process (Lippincott-Schwartz and Phair, 2010; Tu and Banfield, 2010) and that vesicular transport, organelle maturation, and continual fusion and fission of compartments are all likely involved in attaining the steady-state distribution of GTs that ultimately determines cell wall structure. In addition, the formation of large multiprotein complexes can contribute not only to the functioning of those synthetic complexes but also possibly to their retention in the Golgi stacks and distribution across different cisternae (Opat et al., 2000).

The mechanisms of protein endomembrane trafficking in plants have been the subject of intensive studies in the last decade (Stefano et al., 2013; Brandizzi, 2018; Sinclair et al., 2018). Currently, limited information is available about the trafficking of polysaccharide-synthesizing GTs in plant cells (van de Meene et al., 2017). Most studies were devoted to understanding the delivery of CesA proteins and their complexes to the plasma membrane (Watanabe et al., 2018; Zhu et al., 2018). It was suggested that CSCs are pre-formed in the Golgi before being translocated to the cell surface; however, it is unclear whether this is a requirement for their delivery to the plasma membrane. On the other hand, other polysaccharide-synthesizing GTs have never been detected on the plasma membrane and are believed to be retained within the Golgi compartment (Parsons et al., 2012; Rosquete et al., 2018; Okekeogbu et al., 2019; Parsons et al., 2019). It was proposed that the formation of large multiprotein complexes among GTs could prevent their packing into the transport vesicles and assist in Golgi retention (Hassinen et al., 2010). There are also some indications that GTs can pre-form complexes before being transported to the Golgi, and their translocation from the ER to the Golgi could depend on the protein-protein interactions (Jiang et al., 2016). Using confocal and immunogold microscopy imaging, the authors showed that two GTs and two mutases involved in xylan biosynthesis in wheat form complexes that can be detected in the ER but finally accumulate in the Golgi. They proposed that one of these proteins, TaGT43-4, acts as a scaffold protein that holds the other proteins in the complex. There are other important protein-protein interactions that control GTs’ translocation from the ER to the Golgi and their distribution within the Golgi cisternae. These interactions include the components of regulated protein transport machinery and usually involve the N-terminal cytosolic tails and TMDs of GTs (Schmitz et al., 2008; Tu et al., 2008; Liu et al., 2018; Schoberer et al., 2019). Currently, it is unclear whether such interactions could impact the formation of GT complexes and potentially their translocation. However, this area of research is quickly developing and undoubtedly will continue to contribute to our understanding of how GTs get moved from their place of synthesis to their final locations and how and when they are brought together into multiprotein complexes.

## Conclusions and Future Directions

In this review, we have presented the current state of knowledge about plant polysaccharide-synthesizing GTs and the most recent findings on their organization in multiprotein complexes that are required to form the highly branched structures of heteropolysaccharides. There are still significant gaps in our understanding of the stoichiometry, structural organization, functioning, and substrate specificities of GT complexes involved in any type of glycosylation. Nevertheless, it is already evident that the formation and compositional dynamics of these large complexes is key to producing polysaccharide complexity and structural diversity. In addition to GTs, these complexes can also contain specific glycosidases, membrane-bound enzymes responsible for the interconversion of nucleotide sugars, and also multimembrane-spanning transporter proteins required to deliver nucleotide sugars from the cytoplasm (not described in this review). In addition, different acetyl- and methyltransferases can also be a part of the synthesizing complexes (Caffall and Mohnen, 2009; Gille and Pauly, 2012).

New developments in technology and methodologies enable advances in this exciting area of fundamental research. The demonstration of specific protein-protein interactions is an important first step in determining the potential complex formation, but it should always be followed by detailed studies of structural organization of the putative multiprotein complexes and their biological significance. Such detailed studies should include the determination of substrate specificities, the channeling of substrate to specific members of the complex, and the targeting of GTs to particular sub-Golgi compartments and consequently to specific polysaccharide-synthesizing complexes. In addition to the broadly used BiFC and FRET/fluorescence-lifetime imaging microscopy techniques, a recently developed approach in detecting protein-protein interactions using split-biotin assays (Lin et al., 2017; Schopp et al., 2017) enables studies of transient protein interactions *in vivo*, which will certainly advance investigations of GT complex compositional dynamics within different Golgi cisternae and their stability. Novel advances in protein crosslinking, in combination with highly sensitive mass spectrometry techniques (Yang et al., 2017), could also improve our ability to pull down protein complexes formed *via* weak interactions and investigate their composition and stoichiometry. Significant effort is still needed in the field of structural biology of polysaccharide-synthesizing GTs. Without knowledge of diverse GT 3D structures, it is difficult to envision significant progress in understanding the actual structural organization of these complexes, particularly when the complex carries multiple components. Structural studies of GTs using X-ray crystallography and NMR have become more feasible since an expression system using HEK293 cells was introduced for producing recombinant plant GTs (Amos and Mohnen, 2019). The structures of the catalytic domains of the two plant polysaccharide-synthesizing GTs that are currently available were obtained using HEK293 cells. Plant polysaccharide-synthesizing GTs grown in HEK293 cells are capable of producing recombinant proteins in quantities of hundreds of milligrams per liter and secreting them into media. The development of powerful cryo-electron microscopy techniques is opening amazing possibilities in determining structures of large single-component and multicomponent systems and their structural dynamics, with single bond resolution. This approach allowed the discovery of the structure of the CesA trimer involved in cellulose synthesis in plants (Purushotham et al., 2020) and, undoubtedly, will be a pioneering approach in the near future for structural studies of Golgi GT complexes as it overcomes the limitations in studying large membrane proteins and systems with other biophysical techniques.

Ultimately, the understanding of how GTs are organized to synthesize large complex polysaccharide structures will have a significant impact on many aspects of these enzymes’ utilization in plant biotechnology and material science. Thus, the understanding of what brings specific GTs together to synthesize particular structures and how GT activity, substrate specificity, and the structure of their products depend on GT organization in complexes will assist in engineering plant cell wall modifications to increase plant resilience to environmental cues and improving biomass properties for industrial applications. Furthermore, the understanding of GTs’ interdependence will contribute to their application in synthesizing new biomaterials, substituting or complementing chemical synthesis, which currently has significant limitations in synthesizing large, highly branched polysaccharide structures with high regiospecificity.

## Data Availability Statement

The original contributions presented in the study are included in the article/supplementary material, further inquiries can be directed to the corresponding author.

## Author Contributions

OAZ wrote the review. NZ and RW contributed to writing and searching literature. WR prepared the figures. All authors contributed to the article and approved the submitted version.

### Conflict of Interest

The authors declare that the research was conducted in the absence of any commercial or financial relationships that could be construed as a potential conflict of interest.
