# Corrigendum: Polysaccharide Biosynthesis: Glycosyltransferases and Their Complexes

**DOI:** 10.3389/fpls.2021.720709

**Published:** 2021-07-06

**Authors:** Olga A. Zabotina, Ning Zhang, Richard Weerts

**Affiliations:** Roy J. Carver Department of Biochemistry, Biophysics and Molecular Biology, Iowa State University, Ames, IA, United States

**Keywords:** polysaccharide biosynthesis, glycosyltransferases, protein complexes, plant Golgi, structural organization

The name of the second author “Ning Zhang” was incorrectly spelled as “Ning Zang” in the article when published originally.

The authors apologize for this error and state that this does not change the scientific conclusions of the article in any way. The original article has been updated.

